# Human Stanniocalcin-1 Suppresses Angiotensin II-Induced Superoxide Generation in Cardiomyocytes through UCP3-Mediated Anti-Oxidant Pathway

**DOI:** 10.1371/journal.pone.0036994

**Published:** 2012-05-31

**Authors:** Dajun Liu, Luping Huang, Yanlin Wang, Wei Wang, Xander H.T. Wehrens, Tatiana Belousova, Maen Abdelrahim, Gabriel DiMattia, David Sheikh-Hamad

**Affiliations:** 1 Division of Nephrology/Department of Medicine, Baylor College of Medicine, Houston, Texas, United States of America; 2 Department of Molecular Physiology and Biophysics, Baylor College of Medicine, Houston, Texas, United States of America; 3 Cancer Research Institute, MD Anderson Cancer Center Orlando, Orlando, Florida, United States of America; 4 Departments of Oncology and Biochemistry, The University of Western Ontario, London, Ontario, Canada; University of Western Ontario, Canada

## Abstract

**Rationale:**

We have previously shown increased cardiac stanniocalcin-1 (STC1) in patients with idiopathic dilated cardiomyopathy. STC1 localizes to the inner mitochondrial membrane and transgenic over-expression of STC1 is associated with increased energy utilization.

**Objective:**

We examined the hypothesis that STC1 uncouples mitochondrial oxidative phosphorylation - to suppress superoxide generation and modulate neurohormonal effects on cardiomyocytes.

**Methods and Results:**

Compared to WT mouse heart, STC1 Tg heart displays: 2-fold higher uncoupling protein 3 (UCP3) levels, but no effect on UCP2 protein; 40% lower ATP levels; but similar activities of respiratory chain complexes I-IV. In cultured adult rat and freshly-isolated mouse cardiomyocytes, rSTC1 induces UCP3, but not UCP2. Treatment of cardiomyocytes with STC1 decreases mitochondrial membrane potential and suppresses baseline and angiotensin II (Ang II)-induced superoxide generation. Furthermore, baseline superoxide generation is higher in freshly-isolated adult UCP3^−/−^ mouse cardiomyocytes compared to WT, suggesting an important role for UCP3 in regulating cardiomyocyte ROS under physiologic conditions. Treatment of UCP3^−/−^ cardiomyocytes with rSTC1 failed to suppress superoxide generation, suggesting that the effects of STC1 on superoxide generation in cardiomyocytes are UCP3-dependent.

**Conclusion:**

STC1 activates a novel anti-oxidant pathway in cardiac myocytes through induction of UCP3, and may play an important role in suppressing ROS in the heart under normal physiologic conditions and ameliorate the deleterious effects of Ang II-mediated cardiac injury. Importantly, our data point to a critical role for the mitochondria in regulating ROS generation in response to Ang II.

## Introduction

Previous data from our laboratory demonstrated upregulation of the mammalian homologue of fish STC1 in the hearts of patients suffering from idiopathic dilated cardiomyopathy (DCM) [1]. However, the physiologic/pathophysiologic significance of STC1 upregulation in DCM remains to be elucidated. Mammalian STC1 is expressed in many tissues and organs including the heart and skeletal muscle [2] and is defined as an intracrine protein; i.e., an intracellular-acting, extracellular signaling protein [3]. STC1 is released to the extracellular milieu [4] followed by binding to a cell-surface protein [5], internalization into the cell and targeting to the mitochondria [6], [7]; however, the mechanism of internalization and targeting remains unclear. Moreover, recent reports suggest that mammalian STC1 is blood borne [8], [9] – attached to a soluble protein [8], and thus, may also function as a circulating hormone.

Data from McCudden et al. [6] suggested the involvement of STC1 in cellular energy metabolism. Electron microscopy and immunofluorescence studies localized STC1 to the mitochondria [6], [10] and studies in bovine heart mitoplasts suggested that STC1 enhances NADH disappearance [6]; while treatment of isolated mitochondria with STC1 uncoupled phosphorylation [11].

Of interest, STC1 Tg mice are hyperphagic and hypermetabolic [12], [13] and display nearly identical phenotype to UCP3 Tg mice [14]. UCP3 is a homologue of UCP1, which is abundantly expressed in mitochondria of brown adipocytes, where it uncouples phosphorylation to produce heat [thermoregulation] [15]. On the other hand, UCP1 homologues are expressed at low abundance in other tissues and organs [UCP2 in lymphoid cells [16]; UCP3 in the heart and skeletal muscle [17], [18]; UCP4 and UCP5 in the brain [19]] and appear to play an important role in decreasing mitochondrial superoxide generation at the expense of slight reduction in the efficiency of ATP generation [19]–[21]. Therefore, we hypothesized that STC1 induces mitochondrial uncoupling proteins in cardiomyocytes to reduce superoxide generation. Our data confirm this hypothesis and suggest the existence of a novel STC1-induced and UCP3-mediated pathway to suppress baseline and Ang II-induced superoxide generation in cardiomyocytes and point to a critical role for the mitochondria in regulating ROS generation in response to Ang II.

## Results

### Upregulation of Cardiac UCP3 in STC1 Tg Mice

**Figure 1:**
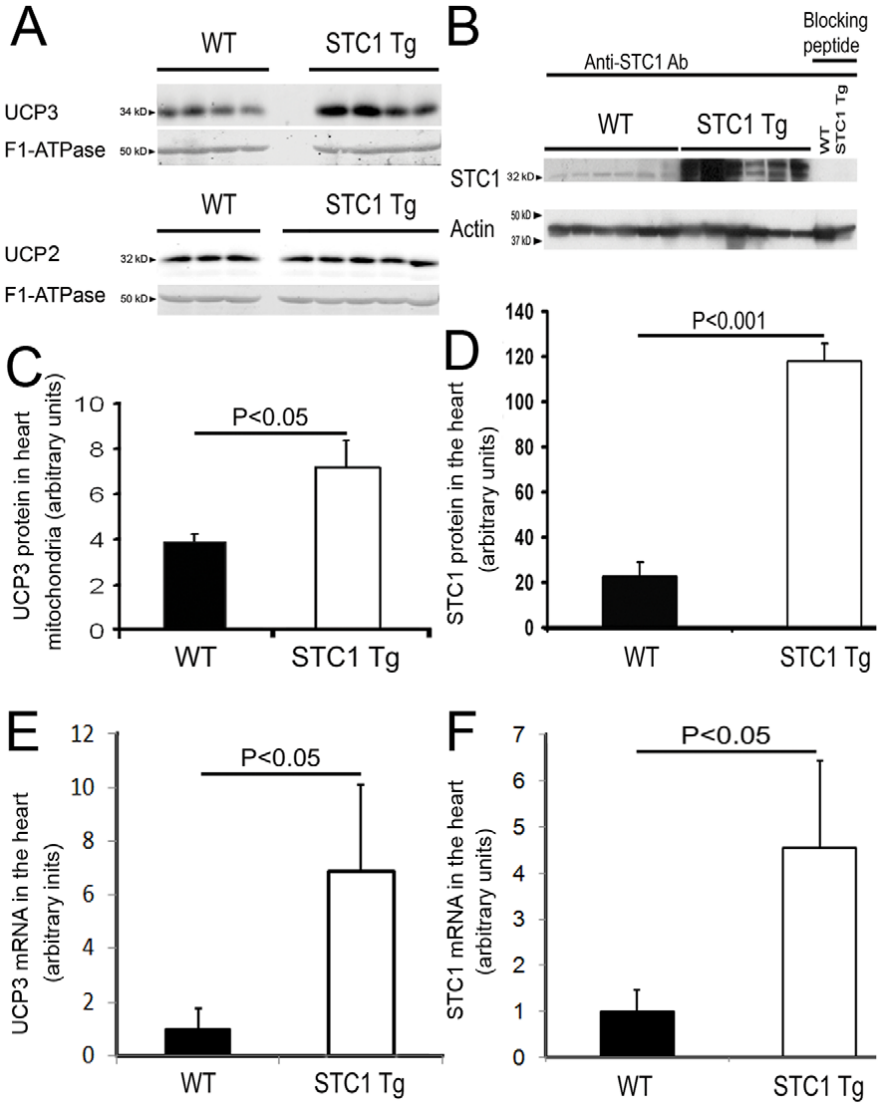
The expression of STC1 positively correlates with UCP3, but not UCP2 in the heart; rSTC1 induces UCP3, but not UCP2 in freshly-isolated mouse cardiac myocytes. I. Increased expression of UCP3, but not UCP2 protein in cardiac mitochondria of STC1 Tg mice. Protein lysates representing ventricular mitochondrial lysates from WT and STC1 Tg mice were resolved on 12% SDS-PAGE, and blots were reacted with anti-UCP3, anti-UCP2 and anti-mitochondrial F1-ATPase (as internal control). Inset (**A**) shows representative Western blots of UCP3, UCP2 and the mitochondrial F1-ATPase. Bar graph (**C**) depicts UCP3 protein levels in heart mitochondrial lysates of WT and STC1 Tg mice, normalized to F1-ATPase protein levels, and presented as arbitrary density units; data represent the mean ± SEM of 4–6 independent determinations from each group. **II. Higher STC1 protein levels in ventricular lysates of STC1 Tg mice:** Protein lysates representing ventricular tissue from WT and STC1 Tg mice were resolved on 12% SDS-PAGE, and blots were reacted with anti-STC1 antibody and anti-β-actin antibody; note the disappearance of STC1 band(s) in the presence of blocking peptide, indicating specificity of the antibody (**B**). Inset (**B**) shows representative Western blots of STC1 and β-actin. Bar graph (**D**) shows band intensities (corresponding to B) normalized to β-actin and presented as arbitrary density units; data represent the mean ± SEM of 6 independent determinations. **III. Correlation between STC1 and UCP3 mRNAs in the heart:** Real-time PCR was carried out on total RNA isolated from WT and STC1 Tg hearts to quantitate STC1 and UCP3 mRNAs as described in methods. Bar graphs depict UCP3 (**E**) and STC1 (**F**) mRNA levels normalized to β-actin mRNA level and presented as arbitrary density units; data represent the mean ± SEM of 3–6 independent determinations.

To determine whether the expression of cardiac STC1 correlates with the expression of UCP2 and UCP3, the prevailing UCPs in the heart [18], we compared whole ventricular or mitochondrial lysates from WT and STC1 Tg mice and found higher level expression of UCP3 protein in mitochondria of STC1 Tg mouse heart compared with WT mice (**Figures 1A and 1C**), that correlated positively with STC1 protein levels in ventricular tissue of STC1 Tg mice (**Figures 1B and 1D**). Similarly, mRNAs for STC1 and UCP3 were higher in STC1 Tg hearts compared with WT hearts (**Figures 1E and 1F**). Quantitatively, and relative to WT, STC Tg hearts displayed 5-fold higher levels of both STC1 mRNA and protein; on the other hand UCP3 mRNA levels were 6-fold higher, while UCP3 protein levels were only 2-fold higher; or 1∶1 correlation between STC1 mRNA and protein *vs* 3∶1 for UCP3 mRNA and protein, respectively, suggesting that while there is positive correlation between STC1 and UCP3, both at the mRNA and protein level, the regulatory schemes for these two genes differ. UCP2 protein levels were similar in mitochondrial lysates of WT and STC1 Tg hearts (**Figures 1A**). These data suggest a role for STC1 in the regulation of cardiac UCP3 and hence, free radicals production in the heart.

### Tg Overexpression of STC1 is Associated with Decreased Cardiac ATP Content, but not the Activities of Respiratory Chain Complexes I-IV

**Figure 2:**
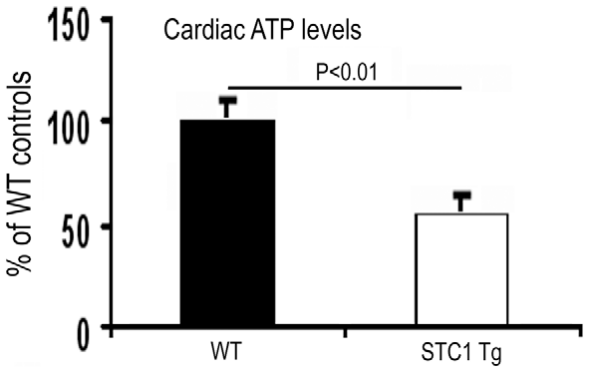
Lower ATP levels in ventricular lysates of STC1 Tg mice compared with WT. Total cellular ATP was extracted from ventricular lysates of WT and STC1 Tg mice and measured using luciferase assay. Values were calculated per gram tissue and presented as % of controls. Data represent the mean ± SEM of 3–6 independent determinations.

Increased expression of UCP3 in cardiac mitochondria is expected to induce uncoupling of phosphorylation; consequently, the efficiency of ATP generation should be lower. In the following experiment, we determined ATP levels in the hearts of WT and STC1 Tg mice. Our results revealed 40% lower ATP levels in ventricular lysates of STC1 Tg mice (**Figure 2**). The data are consistent with increased mitochondrial uncoupling in the heart of STC1 Tg mice and suggest an important role for STC1 in cardiac energy metabolism.

**Figure 3:**
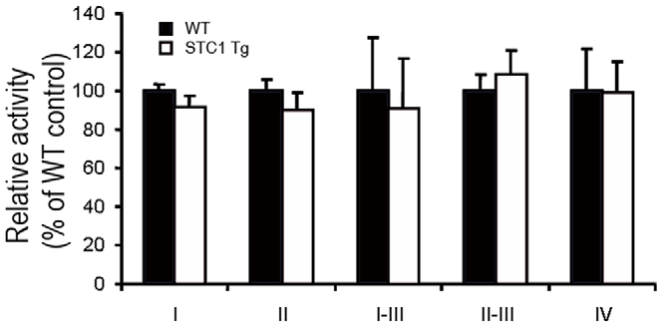
Transgenic overexpression of STC1 does not affect the activities of respiratory chain complexes I-IV in ventricular lysates. Ventricular lysates from WT and STC1 Tg mice were analyzed for activities of respiratory chain complexes as described in methods. Data represent the mean ± SEM of 3–6 independent determinations. Differences were not statistically significant.

Since oxidative phosphorylation and the activities of mitochondrial respiratory chain complexes motivate cellular ATP production, we examined the activities of respiratory chain complexes ***I-IV*** in total heart lysates of WT and STC1 Tg mice. Our results showed no differences in the activities of complex I, complex II, complex I+II, complex II+III, or complex IV (**Figure 3**). These data rule out inhibition of mitochondrial respiratory chain complexes I-IV as the cause for the lower ATP levels we observe in the hearts of STC1 Tg mice.

### STC1 Induces UCP3 in Cardiomyocytes

**Figure 4:**
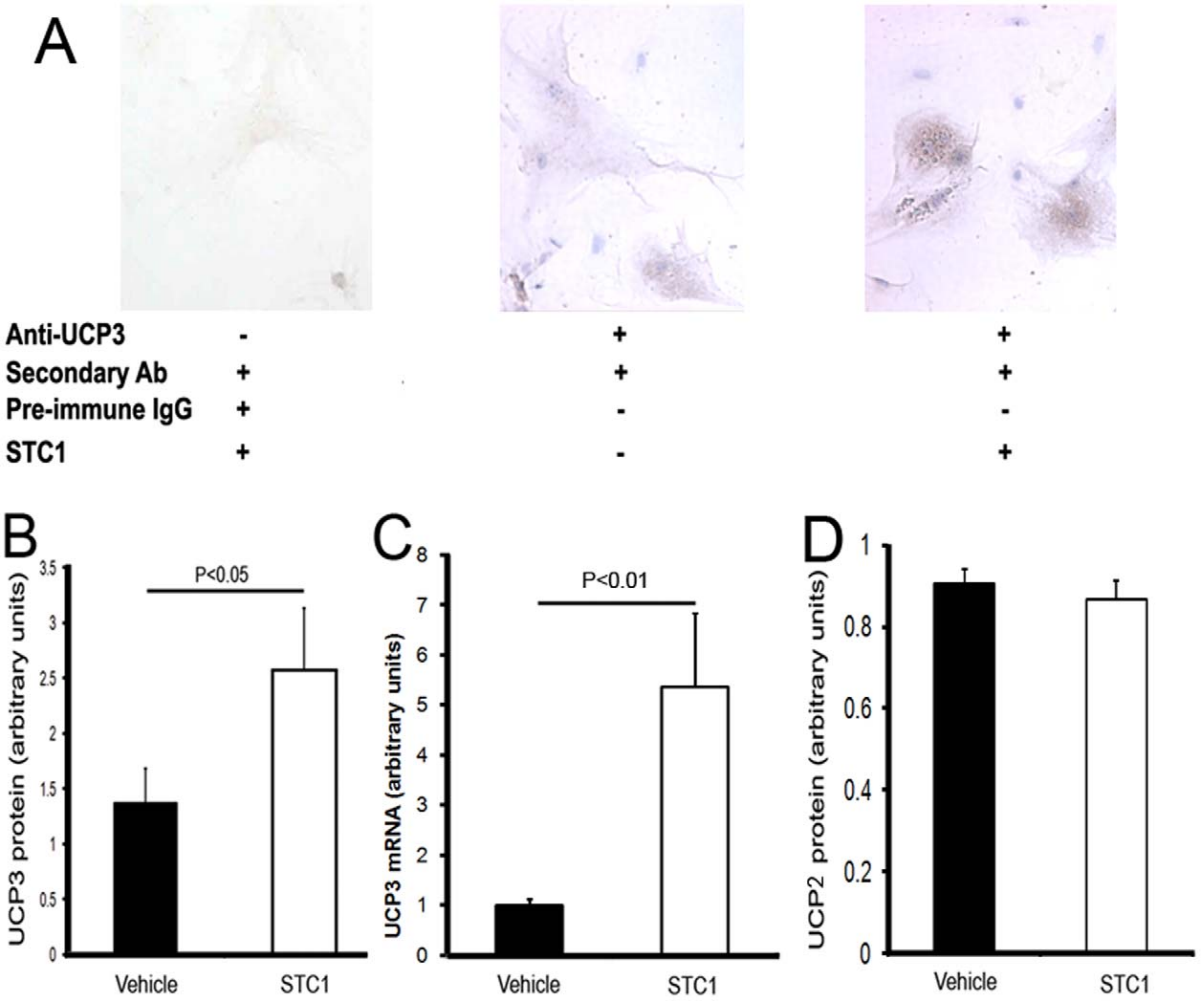
STC1 induces UCP3, but not UCP2 protein in cultured adult rat and freshly-isolated adult mouse cardiac myocytes. A. Cultured primary adult rat cardiac myocytes (7 days after suspension) were treated with rSTC1 protein (100 ng/mL) for 3 h, fixed in 4% saline buffered paraformaldehyde, and stained for UCP3. **B.** Freshly-isolated adult mouse cardiac myocytes were treated with rSTC1 (100 ng/mL) for 3 h, harvested by centrifugation and lysed in RIPA buffer or TRizol (for RNA preparation). Proteins were resolved on SDS-PAGE and Western blots were reacted with anti-UCP3, anti-UCP2 or anti-β-actin antibodies as internal control, followed by standard detection using chemiluminescence. Real-time PCR was carried out on total RNA to quantitate UCP3 mRNA as described in methods. Bar graphs in **B** and **D** depict band intensities for UCP3 and UCP2 proteins, respectively, normalized to β-actin and represent the mean ± SEM of 5 independent determinations. Bar graph in C, depicts UCP3 mRNA, normalized to β-actin mRNA and represent the mean ± SEM of 3 independent determinations.

Uncoupling proteins UCP2 and UCP3 play an important role in regulating superoxide generation [20] and are expressed in the heart [18]. In the following experiment, we sought to determine the effect of rSTC1 protein on the expression of UCP2 and UCP3 in cultured primary adult rat cardiac myocytes and freshly-isolated adult mouse cardiomyocytes. Of note, STC1 levels in human left ventricular tissue obtained from patients with DCM at the time of left ventricular assist device placement may exceed 200 ng/g wet weights (unpublished observations). Treatment of cultured rat cardiomyocytes with STC1 [100 ng/mL; based on dose response curves (data not shown] increased the expression of UCP3 (**Figure 4A**), but had no effect on UCP2 protein expression (data not shown). Similarly, treatment of freshly-isolated adult mouse cardiac myocytes with rSTC1 increased the expression of UCP3 mRNA 5.4-fold (**Figure 4C**), and protein 2-fold (**Figure 4B**), but had no effect on the expression of UCP2 protein (**Figure 4D**).

**Figure 5:**
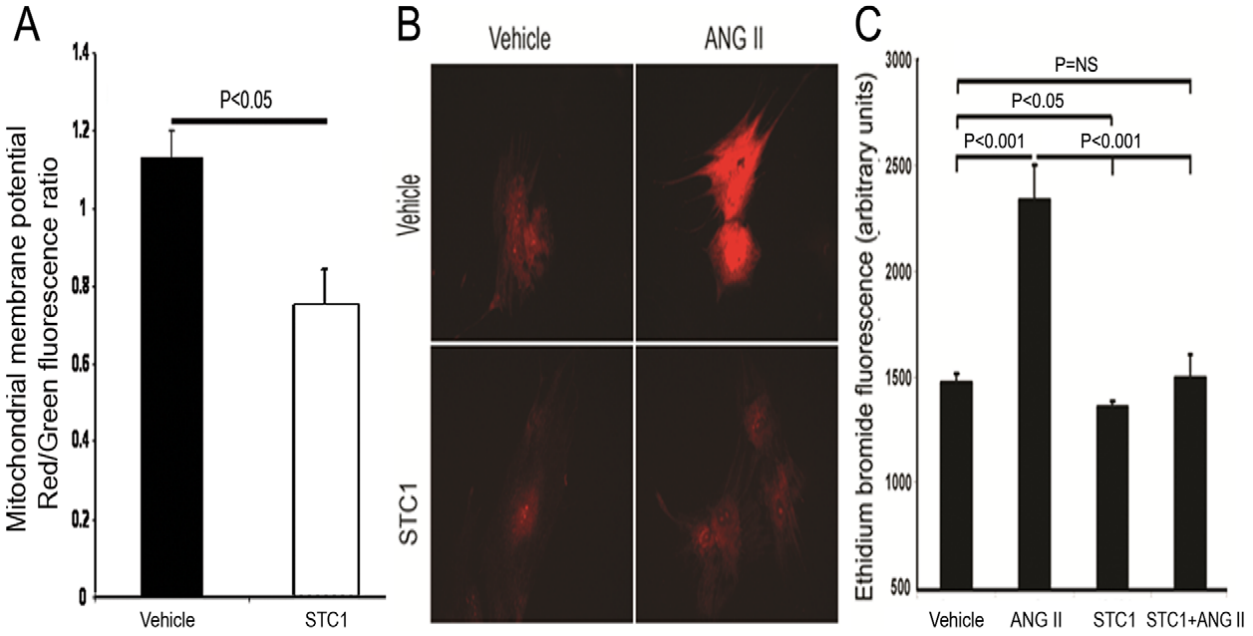
STC1 attenuates mitochondrial membrane potential and diminishes baseline and Ang II-mediated superoxide generation in cardiac myocytes. **A**. Freshly-isolated adult rat cardiac myocytes were treated with rSTC1 protein (100 ng/mL) for 5 h then JC-1 florescence based assay was carried out. Mitochondrial membrane potential (Red/Green fluorescence ratio) was measured, and data represent mean ± SEM of three independent experiments. **B**. Cultured primary adult rat cardiomyocytes (7 days after suspension), were treated for 3 h with PBS, rSTC1 (100 ng/mL), Ang II (100 ng/mL), or Ang II + rSTC1 at the above concentrations. DHE was added to the cell culture during the last 15 min of incubation. After wash with PBS, ethidium bromide fluorescence intensity was measured using fluorescence microscope employing the same settings for all measurements. Representative fluorescence images acquired from cardiomyocytes, treated as above are shown. **C**. Bar graph depicts mean ± SEM of ethidium bromide fluorescence acquired from cardiomyocytes, treated as above (n = 20–40).

Upregulation of uncoupling proteins is expected to shunt protons from the mitochondrial inter-membrane space to the matrix and diminish mitochondrial membrane potential (depolarization). Treatment of cardiac myocytes with rSTC1 (100 ng/mL) for 5 h decreased membrane potential 38% (**Figure 5A**), measured as JC-1 fluorescence. Our data are consistent with STC1-induced and UCP3-mediated mitochondrial uncoupling, and predict attenuation of superoxide generation.

Transient uncoupling of mitochondrial oxidative phosphorylation using pharmacologic uncouplers results in depolarization of mitochondrial membrane potential, diminishing ROS generation [22]–[24]. Hence we examined the effect of rSTC1 (100 ng/mL) on superoxide generation in cultured adult rat cardiac myocytes (measured semi quantitatively as ethidium bromide fluorescence) and determined the impact of STC1 on Ang II-mediated superoxide generation. Of interest, rSTC1 diminishes baseline and Ang II-mediated superoxide generation (**Figure 5B and 5C**), suggesting a potential role for STC1 in regulating cardiac free radicals under physiologic and pathophysiologic conditions.

**Figure 6:**
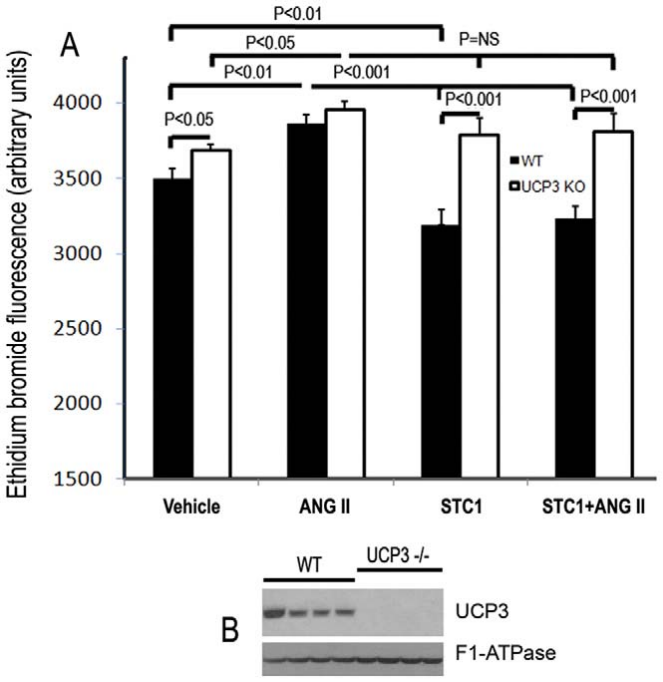
Suppression of superoxide generation by STC1 is UCP3-dependent. Freshly-isolated adult cardiac myocytes from WT and UCP3^−/−^ mice were treated for 3 h with PBS, rSTC1 (100 ng/mL), Ang II (1 µg/mL), or rSTC1+ Ang II at the same concentrations. DHE was added to the medium during the last 15 min of incubation. After wash with PBS, ethidium bromide fluorescence intensity was measured in individual cells using fluorescence microscope employing the same settings for all measurements. **A**. Bar graph depicts mean ± SEM of ethidium bromide fluorescence in vehicle-, Ang II-, rSTC1-, or rSTC1+ Ang II-treated cardiomyocytes (n = 20–40). **B**. Western blot demonstrates absence of UCP3 protein in heart mitochondrial lysates obtained from UCP3^−/−^ mice, compared to WT controls (4 independent determinations). F1-ATPase was utilized as an internal control.

In the following experiments, we sought to determine whether the effects of STC1 on superoxide generation are mediated through uncoupling proteins. Cardiomyocytes express 2 uncoupling proteins, UCP2 and UCP3 [18]. While UCP3 was induced by STC1, UCP2 was not. Therefore, we carried out the next set of experiments using freshly-isolated WT and UCP3^−/−^ mouse cardiomyocytes. In WT cardiomyocytes, STC1 diminishes baseline and Ang II-induced superoxide generation (**Figure 6**), validating our observations in cultured primary rat cardiomyocytes shown in **Figure 5**. Of interest, baseline superoxide generation was higher in UCP3^−/−^ mouse cardiomyocytes, compared to WT (**Figure 6**), and treatment of UCP3^−/−^ cardiomyocytes with STC1 failed to suppress baseline or ANG-II-induced superoxide (**Figure 6**). These data suggest that UCP3 plays an important role in suppressing free radicals in the heart under physiologic conditions, and that suppression of superoxide generation by STC1 is UCP3-mediated. More importantly, our data suggest that suppression of Ang II-mediated superoxide generation by STC1 requires UCP3, pointing to a critical role for the mitochondria in regulating ROS generation in response to Ang II. Collectively, our data suggest the existence of a novel STC1-induced and UCP3-mediated pathway to suppress baseline and Ang II-induced superoxide generation in cardiomyocytes.

## Discussion

The function of mammalian STC1 is not fully defined. Previous data from our lab suggested a role for STC1 in cardiac function, as the expression of STC1 is markedly increased in cardiomyoctes and blood vessels of failing human heart secondary to DCM [1]; whereas, following treatment with left ventricular assist device, STC1 expression is “normalized” [1], suggesting regulation of STC1 by modifiable factors related to ventricular load. However, it was unclear what role STC1 plays in DCM. Several lines of evidence implicate ROS in the development and progression of heart failure (reviewed in [25]): first, the levels of ROS are elevated in both plasma and myocardial tissue in heart failure; second, hypertrophy and apoptosis of cardiac cells in response to neurohormones such as norepinephrine, ANG-II, or cytokines appear to be modulated by redox-sensitive pathways, as the administration of scavengers of ROS diminishes cardiac remodeling after myocardial infarction, a condition characterized by increased generation of ROS. Mitochondrial electron transport chain and non-phagocytic NADPH oxidases are the predominant sources of ROS in cardiomyocytes [26], [27]; however little is known about the contribution and regulation of specific sources of ROS in response to neurohormones.

Heart failure is associated with activation of the renin-angiotensin-system and it is well-established that ANG-II induces cardiac injury partially through increased generation of ROS [25], [28]. Recent data suggest an important role for uncoupling proteins in the regulation of ROS in the heart and other organs. Since STC1 is upregulated in the heart in patients suffering from dilated cardiomyopathy, we reasoned that STC1 may play a role in suppressing Ang II-mediated ROS. Hence, we examined the effect of STC1 on Ang II-induced superoxide generation in cultured adult rat cardiomyocytes and freshly-isolated adult UCP3^−/−^ mouse cardiac myocytes. We chose UCP3−/− cardiomyocytes because UCP3 is the predominant UCP in the heart [18], was increased in STC1 Tg hearts and was induced by STC1 in cultured rat cardiomyocytes and freshly-isolated mouse cardiac myocytes, while UCP2 was not. Our data suggest that: A) STC1 decreases baseline and Ang II-induced superoxide generation in cardiac myocytes; B) suppression of superoxide by STC1 is UCP3-dependent; C) knockout of cardiomyocyte UCP3 is associated with increased baseline superoxide generation, suggesting an important role for UCP3 in suppressing free radicals production in the heart under normal physiologic conditions. Finally, STC1 fails to suppress Ang II-mediated superoxide generation in the absence of UCP3 expression.

The above data suggest that STC1 may have a protective role in DCM, and may suppress ANG-II-mediated superoxide generation in cardiomyocytes in vivo. STC1 action appears to require mitochondrial UCP3 expression and importantly, our data point to a critical role for the mitochondria in regulating ROS in response to Ang II. It was traditionally thought that uncoupling of the mitochondria increases the generation of ROS. However, considerable recent evidence suggests otherwise. A group of UCP1 homologues has been discovered recently [UCP2 in the lymphoid system; UCP3 in the heart and skeletal muscle [17], [18]; UCP4 and UCP5 in the brain [19]], and like pharmacologic uncouplers [24], they diminish superoxide generation [19]–[21]. Therefore, increased expression of UCP3 in cardiomyocytes is expected to suppress the generation of ROS at the expense of slight reduction in the efficiency of ATP generation (reviewed in [29]). In vivo, we find positive correlation between the levels of STC1 and UCP3 protein in the heart. Consistently, ATP levels were lower in the heart of STC1 Tg mice compared to WT mice. However, the activities of respiratory chain complexes I-IV were similar. These data suggest that the lower ATP levels we observe in the hearts of STC1 Tg mice are related to mitochondrial uncoupling, rather than to inhibition of respiratory chain.

The cellular and molecular mechanisms underlying DCM are unclear. DCM may result from inherited defects in mitochondrial energy metabolism; but, may also be associated with acquired transcriptional dysregulation of metabolic enzymes [30]–[32], and changes in mitochondrial oxidative phosphorylation [33]. Thus, changes in mitochondrial structure/function may underlie DCM and/or play a role in the adaptive/compensatory responses initiated by the primary disease process. Low levels of ATP have been reported to occur in the hearts of patients suffering from DCM [34], [35] and considering the higher levels of STC1 in the hearts of DCM patients [1]. It is tempting to speculate that the lower cardiac ATP levels observed in some of these patients may result from STC1-driven and UCP3-mediated uncoupling of mitochondrial phosphorylation. The “trade off”, of course, is the reduction in the generation of free radicals, which is expected to provide cardio protection. While deletion of UCP3 increases ROS [36], the long-term effects of UCP3 upregulation in the heart are unknown. Knockout of UCP3 increases the efficiency of ATP generation in skeletal muscle without affecting tricarboxylic acid cycle flux rate [37], and it is reasonable to assume that increased expression of UCP3 in the heart would predict reduction in the efficiency of ATP generation and have deleterious effects on cardiac function; however STC1 Tg mice display no overt cardiac phenotype. Moreover, while STC1 inhibits L-type calcium channels in cultured cardiomyocytes [1], an effect that may have a negative impact on myocardial contractility and blood pressure, particularly in the setting of heart failure [38], we observe normal blood pressure in STC1 Tg mice, and unpublished observations from our lab suggest that STC1 Tg mice outlive WT littermates.

In conclusion, our data suggest that STC1 may be an important player in regulating ROS in the heart under normal physiologic conditions and in the adaptive responses to pathophysiologic states associated with DCM. Our data indicate that STC1 may also play a critical role in ameliorating ANG-II-mediated oxidant injury and point to a critical role for the mitochondria in regulating ROS generation in response to Ang II.

## Materials and Methods

### Ethics Statement

The investigation conforms to the Guide for the Care and Use of Laboratory Animals published by the US National Institutes of Health (NIH Publication No. 85-23, revised 1996). Animal experiments were approved by Institutional Animal Care and Use Committee (IACUC).

### Materials

All materials were purchased from Sigma (St Louis, MO) unless stated otherwise. Recombinant hSTC1 protein was kindly provided by Dr. Henrik Olsen, Human Genome Sciences (Rockville, MD). It was produced using a baculovirus expression system and is greater than 90% pure [39]. Endotoxin levels in STC1 preparation were determined using Limulus Amebocyte Lysate Test Kit (Cambrex Bio Science Walkersville, MD) according to manufacturer’s instructions, and showed no detectable endotoxin. Goat anti-hSTC1 antibodies, immunizing peptide for STC1, and goat anti-F1-ATPase were purchased from Santa-Cruz (Santa Cruz, CA). Goat anti-UCP2 antibodies were purchased from LifeSpan Bioscience Inc. (Seattle, WA). Rabbit anti-UCP3 antibodies were purchased from Affinity Bioreagents (Golden, CO).

### Genetically-modified Mice

STC1 Tg mice were generated by Varghese et al. [12] and made available for these studies. STC1 transgene expression is driven by the metallothionein I minimal promoter and displays preferential expression in the liver, brain, heart, and endothelial cells [9], [12]. Tg mice have normal blood pressure [9] and display no overt cardiac phenotype. Serum calcium is normal, while serum phosphate levels are slightly elevated [12]. Kidney function and hematocrit are also normal (Kidney International, in press). UCP3 null mice were a gift from Dr Edward Mills, University of Texas, Austin, and have been described previously [40]. All studies were carried out using 4–6 mo old mice homozygous for the transgenes on C57BL/6 genetic background and the respective WT mice generated from crosses between mice heterozygous for the transgene.

### Preparation of Cultured Adult Rat Cardiomyocytes

Adult rat cardiomyocytes were harvested as previously described [41], [42]. Briefly, following anesthesia (combination anesthetic contains/1 mL: ketamine 37.5 mg, xylazine 1.9 mg and acepromazine 0.37 mg; given at 0.75–1.5 mL/kg body weight), rats (4–6 mo old; average weight of 200 g) were given 3000 IU of heparin by I.V. injection. The heart was exposed by a longitudinal thoracotomy incision, and the thymus and fascia were cleared from the aorta with a sterile swab. The aorta was cross-clamped and cut distally, and the heart was removed and placed in 50 mL of Joklik media [1 package of Joklik media powder (Gibco/BRL), suspended in 50 mL water, and supplemented with 3.91 g taurine, 2.0 g NaHCO_3_, 0.391 g L-glutamine and 0.282 g adenosine]. The heart was rinsed and transferred to fresh ice-cold Joklik media. The aorta was cannulated and flushed with cold Joklik media using a syringe, followed by perfusion with a perfusion pump at a rate of 12–15 mL/min, for 5 minutes. The heart was then transferred to a Langendorff apparatus, flushed with warm Joklik media, and digested by perfusion with Joklik media containing 0.1% collagenase and 0.1% trypsin, for 45 minutes.

The ventricles were minced and placed in digestion buffer containing 0.1% collagenase (in Joklik media). Minced heart tissue was incubated in a shaking water bath at 37°C for 30 minutes. The supernatant was transferred to a conical tube and centrifuged (for 3 minutes, at 50 g). The resultant pellet was washed twice in 4% BSA solution and once in 2% BSA solution. The pellet was then suspended in 20 mL Joklik media (pH 7.2), containing 2% BSA, followed by slow addition of CaCl_2_ to yield a final concentration of 1.25 mmol/L. The cells were finally recovered by centrifugation (as above) and suspended by gentle pipetting in 4 mL of warm, serum-free DMEM.

The cell suspension (1–2 drops at a time) was then layered onto sterile laminin-coated cover slips. After 30 minutes of incubation at 37°C in 5% CO_2_/95% O_2_ (to allow cell attachment) plating media [DMEM containing 10% fetal bovine serum, 3 µg/mL cytarabine (to inhibit fibroblast growth), 10 µg/mL insulin, and 5 U/mL each of penicillin & streptomycin] was gently added. Cells were fed with fresh plating media every other day. The cells began beating after 5–7 days of incubation, and the experiments were carried out between days 7–14.

### Preparation of Freshly-isolated Mouse Cardiomyocytes

Mouse ventricular myocytes were isolated as described previously [43]. Briefly, the heart was removed following 1% isoflurane anesthesia and rinsed in 0 Ca^2+^ Tyrode solution (137 mmol/L NaCl, 5.4 mmol/L KCl, 1 mmol/L MgCl_2_, 5 mmol/L HEPES, 10 mmol/L glucose, 3 mmol/L NaOH, pH 7.4). The heart was cannulated through the aorta and perfused on a Langendorff apparatus with 0 Ca^2+^ Tyrode (3 ∼ 5 minutes, 37°C), then 0 Ca^2+^ Tyrode containing 20 **µ**g/mL Liberase TH Research Grade (0.104 a.u./mL; Roche Applied Science) for 10 ∼ 15 minutes at 37°C. After digestion, the heart was perfused with 5 mL KB solution (90 mmol/L KCl, 30 mmol/L K_2_HPO_4_, 5 mmol/L MgSO_4_, 5 mmol/L pyruvic acid, 5 mmol/L β-hydroxybutyric acid, 5 mmol/L creatine, 20 mmol/L taurine, 10 mmol/L glucose, 0.5 mmol/L EGTA, 5 mmol/L HEPES, pH 7.2) to wash out collagenase. The left ventricle of the heart was minced in KB solution and gently agitated, then filtered through 210 µm polyethylene mesh. After settling, ventricular myocytes were washed once with KB solution, and then stored in KB solution at room temperature before use.

### Measurement of Mitochondrial Membrane Potential

Mitochondrial membrane potential was measured using 3,3′-tetraethylbenzimidazolylcarbo-cyanine iodide (JC-1), as per manufacturer’s instructions (Molecular Probe, Eugene, OR). JC-1 is a lipophilic, cationic dye that can selectively enter into mitochondria and reversibly change color from green to red as the membrane potential increases. In cells with high mitochondrial membrane potential, JC-1 spontaneously forms complexes known as J-aggregates emitting intense red fluorescence (at ∼590 nm). On the other hand, in cells with low membrane potential, JC-1 remains in the monomeric form, which emits only green fluorescence (at ∼530 nm). Freshly-isolated adult rat cardiac myocytes were treated with rSTC1 protein or PBS for the indicated times. After treatment, cells were incubated with 50 nmol/L JC-1 at 37°C for 30 min and washed (3X) with PBS (contains 3.2 mmol/L Na_2_HPO_4_, 0.5 mmol/L KH_2_PO_4_, 1.3 mmol/L KCl, 135 mmol/L NaCl, pH 7.4). The red JC-1 fluorescence was measured at 530 nm excitation/590 nm emission, and the green JC-1 fluorescence was measured at 485 nm excitation/530 nm emission using a fluorescence-activated fluorescence reader (BMG Lab Technologies, Germany). After subtraction of background values obtained from wells containing JC-1 but devoid of cells, red/green fluorescence ratios were calculated.

### Semi Quantitative Measurement of Superoxide

Mitochondria have been reported to generate superoxide and may release these radicals into the extra-mitochondrial space [44]. Cultured adult rat cardiac myocytes were treated for 3 h with combinations of the following: STC1 (100 ng/mL; optimal concentration for induction of UCP3); Ang II (100 ng/mL); or vehicle (PBS). During the last 15 min of treatment, cells were incubated with 10 µmol/L dihydroethidium (DHE). After wash with ice-cold PBS, ethidium bromide fluorescence intensity from individual cells was measured using fluorescence microscope employing the same settings for all measurements. Fluorescence data derived from 20–40 cells per treatment were used for analysis.

### Isolation of Heart Mitochondria

Mitochondria were isolated from mouse heart using a trypsin digestion procedure as previously described [45]. Briefly, ventricular tissue was minced, washed, and suspended in 1 mL of isolation medium [0.3 mol/L sucrose, 10 mmol/L HEPES (pH 7.2), and 0.2 mmol/L EDTA]. The tissue was subjected to mild trypsin digestion (0.125 mg/mL) for 15 min at 4°C, followed by the addition of 1 mL isolation medium (pH 7.4) containing 1 mg/mL BSA and 0.65 mg of trypsin inhibitor. The partially digested tissue was suspended in 1 mL of isolation medium containing 1 mg/mL albumin and 1X complete protease inhibitor (Roche diagnostics, Germany) and homogenized briefly with a high viscosity mixer-glass homogenizer (Henry Troemner LLC, USA). The homogenate was centrifuged on a bench-top centrifuge for 10 min at 2000 rpm (4°C). The supernatant was recovered and centrifuged for 15 min at 11000 rpm (4°C). The resulting pellet was washed twice in 1 mL of isolation medium containing 1 mg/mL albumin and 1X complete protease inhibitor. The pellet was recovered each time by centrifugation at 11000 rpm for 15 min (4°C). The final pellet was suspended in 0.1 mL of isolation medium containing 1 mg/mL albumin and 1X complete protease inhibitor. Protein was determined by the Bradford assay using BSA as a standard.

### Measurement of Tissue ATP Level

Tissue ATP content was measured using the bioluminescent somatic cell ATP assay kit (Sigma), as per manufacturer’s instructions. Briefly, freshly harvested ventricular tissue from WT or STC1 Tg mice was homogenized in somatic cellular ATP releasing reagent, and cell lysates were incubated with ATP assay mix. Cellular ATP levels were measured as bioluminescence using a TD-20/20 luminometer (Turner Designs Instruments, Sunnyvale, CA), and data were expressed as % of WT controls.

### Mitochondrial Respiratory Chain Activity Assay

Ventricular tissue homogenates were prepared at 4°C using Teflon homogenizer, in a buffer containing 250 mmol/L sucrose, 2 mmol/L EDTA and 100 mmol/L Tris-HCl, pH 7.4 [46]. The assay was carried out on homogenates at 30°C using a temperature-controlled spectrophotometer (Pharmacia, Biotech; Piscataway, NJ). The activities of mitochondrial respiratory chain **complex I** (NADH dehydrogenase), **complex II** (succinate dehydrogenase), **complex I + III** (NADH:cytochrome c reductase), **complex II + III** (succinate:cytochrome c reductase) and **complex IV** (cytochrome c oxidase) were assayed using different electron acceptors/donors as previously described [47], [48]. NADH dehydrogenase activity was measured as the rate of NADH oxidation (*measurement of NADH absorbance at 340 nm*), using potassium ferric cyanide as the electron acceptor. Succinate dehydrogenase activity was measured as the rate of 2,6-dichlorophenolindophenol (DCIP) reduction (*measurement of DCIP absorbance at 600 nm*), using succinate as electron donor (*reaction is carried out in the presence of potassium cyanide (KCN) – to inhibit cytochrome c oxidase*). NADH:cytochrome c reductase activities were measured as the rate of cytochrome c reduction (*measurement of cytochrome c absorbance at 550 nm*), using NADH as electron donor (*reaction is carried out in the presence of KCN – to inhibit cytochrome c oxidase*). The activities of succinate:cytochrome c reductase were measured as the rate of cytochrome *c* reduction (*measurement of cytochrome c absorbance at 550 nm*), using succinate as electron donor (*reaction is carried out in the presence of KCN – to inhibit cytochrome c oxidase*). Cytochrome c oxidase activity (*measurement of cytochrome c absorbance at 550 nm*) was measured as the rate of oxidation of freshly reduced cytochrome c, using Na hydrosulfate. To adjust enzymatic activities for mitochondrial content, the activities were expressed as percentage of values in controls and normalized to citrate synthase activity - measured as the reaction of sodium oxaloacetate, acetyl-coenzyme A and 5,5′-dithio-bis-(2 nitrobenzoic) acid at 412 nm [48].

### Western Blot Analysis

Heart mitochondrial preparations (to assay UCPs) or total heart lysates (to assay STC1) from WT or STC1 Tg mice [12], suspended in modified RIPA buffer [containing 150 mmol/L NaCl, 50 mmol/L Tris-HCl (pH 7.4), 1% NP-40, 0.25% sodium deoxycholate, 1 mmol/L EDTA, and 1X complete protease inhibitor cocktail] were centrifuged at 8000 g for 10 min at 4°C to remove cell debris. Fifteen µg of mitochondrial protein were resolved on 12% SDS-PAGE, transferred to nitrocellulose membrane and incubated with primary antibodies for UCP2, UCP3 and F1-ATPase as internal controls. Similarly, 50 µg of protein lysate from freshly isolated mouse cardiomyocytes or ventricular tissue were loaded per lane and Western blots reacted with primary antibodies for UCP2, UCP3, STC1 or actin as internal control. After washing with Tris buffered saline [20 mmol/L Tris (pH 7.6), 137 mmol/L NaCl] containing 0.1% Tween-20, the membrane was incubated with horse radish peroxidase-conjugated secondary antibody, and the bound antibodies were visualized using chemiluminescence.

### RNA Isolation/Real-time PCR

Total RNAs was prepared from mouse heart tissues or mouse cardiomyocytes using TRIzol reagent (Invitrogen, Grand Island, NY) according to the manufacturer’s instructions. Total RNA was treated with DNase I (Sigma, St. Louis, MO) prior to cDNA production using cDNA synthesis Kit (Bio-Rad Laboratories, Hercules, CA). Reaction conditions for cDNA synthesis were 25°C for 5 min, 42°C for 30 min and 85°C for 5 min. Real-time PCR was performed using Bio-Rad Real-Time PCR CFX 96 system. Sequences for real-time PCR primers: STC1 forward primer (5′-CCATCACTGAAGTCATACA-3′) and reverse primer (5′-TCATCACATTCCAGAAGG-3′); UCP3 forward primer (5′-CTGTCATCGTCATCATCTA-3′), and reverse primer (5′-GCTTTACCACTACAAACTG-3′); β-actin forward primer (5′- ATCTTCCGCCTTAATACT -3′), and reverse primer (5′-GCCTTCATACATCAAGTT -3′). Twenty five nanograms of total RNA were used for each PCR reaction with SYBR Green (Bio-Rad Laboratories) detection at 10 µl reaction volumes. The reaction conditions for PCR were (*stage 1*, Rep 1x) 95°C for 3 min, (stage 2, Rep 1x) 95°C for 5 s, and (*stage 3*, Rep 39x) 60°C for 30 s and then 60–95°C for 5 s. The relative mRNA expression levels were calculated from cycle threshold (*C_t_*) values using β-actin as the endogenous control (relative expression = 2^(*target* Ct*−* reference *Ct*)^).

### Statistical Analysis

Data were expressed as the mean ± SEM. Statistical significance was determined by unpaired t test or ANOVA as appropriate. A *p* value of <0.05 was considered statistically significant.
