# Correction: Human Stanniocalcin-1 Suppresses Angiotensin II-Induced Superoxide Generation in Cardiomyocytes through UCP3-Mediated Anti-Oxidant Pathway

**DOI:** 10.1371/annotation/52d155b7-66af-425f-9a6a-2d853d5fd95d

**Published:** 2012-09-05

**Authors:** Dajun Liu, Luping Huang, Yanlin Wang, Wei Wang, Xander H.T. Wehrens, Tatiana Belousova, Maen Abdelrahim, Gabriel DiMattia, David Sheikh-Hamad

The equal contributions statement was omitted. Dajun Liu, Luping Huang and Yanlin Wang contributed equally to the manuscript.

